# Inhibition of LINC00958 hinders the progression of osteoarthritis through regulation of the miR-214-3p/FOXM1 axis

**DOI:** 10.1186/s13018-024-04545-x

**Published:** 2024-01-13

**Authors:** Yingchuan Yin, Qiaojuan He, Jing He, Ying Feng, Yunyun Xu

**Affiliations:** Endocrinology Department, The Third People’s Hospital of Hefei, No.204, Wangjiangdong Road, Hefei, 230022 China

**Keywords:** LINC00958, Osteoarthritis, Inflammation, miR-214-3p/FOXM1 axis

## Abstract

**Objective:**

We investigated the impact of the long noncoding RNA LINC00958 on cellular activity and oxidative stress in osteoarthritis (OA).

**Methods:**

We performed bioinformatics analysis via StarBase and luciferase reporter assays to predict and validate the interactions between LINC00958 and miR-214-3p and between miR-214-3p and FOXM1. The expression levels of LINC00958, miR-214-3p, and FOXM1 were measured by qRT-PCR and western blotting. To assess effects on CHON-001 cells, we performed MTT proliferation assays, evaluated cytotoxicity with a lactate dehydrogenase (LDH) assay, and examined apoptosis through flow cytometry. Additionally, we measured the levels of apoptosis-related proteins, including BAX and BCL2, using western blotting. The secretion of inflammatory cytokines (IL-6, IL-8, and TNF-α) was measured using ELISA.

**Results:**

Our findings confirmed that LINC00958 is a direct target of miR-214-3p. LINC00958 expression was upregulated but miR-214-3p expression was downregulated in both OA cells and IL-1β-stimulated CHON-001 cells compared to the corresponding control cells. Remarkably, miR-214-3p expression was further reduced after miR-214-3p inhibitor treatment but increased following LINC00958-siRNA stimulation. Silencing LINC00958 significantly decreased its expression, and this effect was reversed by miR-214-3p inhibitor treatment. Notably, LINC00958-siRNA transfection alleviated the IL-1β-induced inflammatory response, as evidenced by the increased cell viability, reduced LDH release, suppression of apoptosis, downregulated BAX expression, and elevated BCL2 levels. Moreover, LINC00958 silencing led to reduced secretion of inflammatory factors from IL-1β-stimulated CHON-001 cells. The opposite results were observed in the miR-214-3p inhibitor-transfected groups. Furthermore, in CHON-001 cells, miR-214-3p directly targeted FOXM1 and negatively regulated its expression.

**Conclusion:**

Our findings suggest that downregulating LINC00958 mitigates IL-1β-induced injury in CHON-001 cells through the miR-214-3p/FOXM1 axis. These results imply that LINC00958 plays a role in OA development and may be a valuable therapeutic target for OA.

## Introduction

Osteoarthritis (OA), among the most prevalent degenerative joint diseases, is caused by a multitude of factors, including aging, obesity, strain, and trauma [1, 2]. This condition affects more than approximately 240 million individuals worldwide, predominantly middle-aged and elderly people [3]. Crucially, chondrocytes serve as vital protectors of matrix integrity [4]. Presently, the pathogenesis of OA is thought to involve intricate interplay among multiple factors, and effective prevention and treatment methods are lacking. Hence, the search for efficacious OA treatments has become paramount. Emerging evidence implicates various cytokines in cartilage degradation, with IL-1β a prominent player [5]. Notably, Yang et al. investigated the mechanism by which downregulation of microRNA-23b-3p alleviates IL-1β-induced injury in chondrogenic CHON-001 cells [6]. Consequently, we postulate that inhibiting IL-1β expression may be the key to mitigating OA.

Long noncoding RNAs (lncRNAs) are a class of RNA molecules exceeding 200 nucleotides in length. Serving as a pivotal layer in biological regulation, lncRNAs significantly influence various biological processes, including regulation of the cell cycle and cell differentiation [7]. LINC00958, in particular, has been referenced in numerous contexts, across diverse cancers. Zhou et al. [8] described how LINC00958 drives tumour progression through the miR-4306/CEMIP axis in osteosarcoma. Li et al. proposed that the LINC00958/miR-3174/PHF6 axis orchestrates the cell proliferation, migration, and invasion observed in endometrial cancer [9]. However, the specific mechanism of LINC00958 in the context of OA remains to be explored further.

MicroRNAs (miRNAs), a subclass of noncoding RNAs, have garnered substantial recognition as pivotal regulators of diverse cellular processes, through their binding to target mRNAs [10–12]. Similarly, Ding et al. [13] suggested that miR-93 inhibits chondrocyte apoptosis and inflammation in OA through the TLR4/NF-kappaB (κB) signalling pathway. Wang et al. [14] suggested that circATRNL1 protects against OA by targeting miR-153-3p and KLF5. Concurrently, Fioravanti et al. [15] pinpointed miR-214-3p as a promising therapeutic target in the context of OA pathogenesis. Conversely, downregulation of miR-214-3p has been implicated in activating the NF-κB pathway, exacerbating the progression of OA [16]. However, for a comprehensive understanding, further analysis is warranted to elucidate the complete set of functions and molecular mechanisms governed by miR-214-3p in the contest of OA.

Hence, our study was structured to elucidate the roles of LINC00958 in the pathogenesis of OA. In this study, we propsed the following hypotheses: (i) stimulation of CHON-001 cells with IL-1β may accelerate damage in human chondrocytes, cnstituting an in vitro model for studying inflammation; (ii) LINC00958 exhibits a protective effect against OA-related behaviors of CHON-001 cells following IL-1β treatment; and (iii) the underlying mechanisms responsible for the protective effects of LINC00958 could be intricately linked to the miR-214-3p/FOXM1 axis. These findings could lead to the development of a promising effective therapeutic approach for OA.

## Materials and methods

### Cell culture

CHON-001 cells were purchased from the ATCC and grown in DMEM (Thermo Fisher) supplemented with 10% FBS and 1% penicillin–streptomycin under humidified conditions with 5% CO_2_ at 37 °C. Subsequently, CHON-001 cells were stimulated with 10 ng/ml IL-1β for 12 h to establish an in vitro cellular model of inflammatory injury.

### Dual-luciferase reporter assay

To investigate the relationships of miR-214-3p with LINC00958 and FOXM1, StarBase was utilized. We employed the WT-LINC00958 and MUT-LINC00958 3′-UTR luciferase reporter plasmids to clarify the potential interactions between miR-214-3p and LINC00958. Through this approach, LINC00958 was identified as a potential target of miR-214-3p. In the luciferase activity assay, the LINC00958 wild-type or mutant plasmid was co-transfected with the miR-214-3p mimic or mimic control into 293 T cells using Lipofectamine 2000 (Invitrogen) following the provided protocol for a duration of 24 h. Subsequently, the luciferase activity was measured using the dual-luciferase reporter assay system (Promega, USA).

### Cell transfection

To modulate LINC00958 or miR-214-3p expression in CHON-001 cells, we used control-siRNA, LINC00958-siRNA, an inhibitor control, a miR-214-3p inhibitor, a mimic control, or a miR-214-3p mimic. All transfections were performed using Lipofectamine^®^ 3000 reagent (Thermo), following the manufacturer’s instructions, and the cells were incubated for 48 h. Subsequently, we assessed the cell transfection efficiency by qRT-PCR.

### qRT-PCR analysis

We extracted total RNA from CHON-001 cells using TRIzol reagent (TaKaRa, Shiga, Japan) following the manufacturer’s instructions. Subsequently, total RNA was reverse transcribed into cDNA using the PrimeScript RT Reagent Kit (TaKaRa, China). PCR amplification was conducted on an ABI PRISM 7900 sequence detection system (Applied Biosystems, USA) to measure the levels of LINC00958, miR-214-3p, FOXM1, and GAPDH. The expression of target genes was quantified using the 2^−ΔΔCt^ method.

### MTT assay

Following treatment, CHON-001 cells were cultured in 96-well plates at 37 °C. Subsequently, the cells were treated with 10 μl of MTT solution (5 mg/ml) and incubated for an additional 4 h. Following this incubation step, the solution was carefully removed, and 100 μl of DMSO was added to each well in the dark to dissolve the formazan crystals. Finally, after 15 min of gentle mixing, the optical density (OD) at 490 nm was measured using a multifunctional plate reader (BioTek, USA) following the manufacturer’s instructions.

### Flow cytometry (FCM) analysis

We assessed apoptosis in CHON-001 cells using an Annexin-V/Propidium Iodide (PI) Apoptosis Detection Kit (BD Biosciences) with incubation at room temperature for 10 min following the provided instructions. Apoptotic cells were subsequently quantified using a flow cytometer (BD Technologies), and the data were analysed with FlowJo software.

### Western blotting analysis

Proteins were extracted from CHON-001 cells using RIPA buffer (Beyotime), and protein concentrations were measured using a BCA Protein Assay Kit (Invitrogen, USA). Subsequently, proteins in the samples were separated on a 10% SDS‒PAGE gel and then transferred onto a PVDF membrane (Millipore, USA). After blocking with 5% skim milk in PBST for 1 h, the membranes were incubated overnight at 4 °C with primary antibodies against β-actin, Bax, Bcl-2, and FOXM1 (1:1000 dilutions). The membranes were subsequently incubated for 1 h with secondary antibodies. Finally, protein signals were visualized using the ECL method (Cytiva) following the manufacturer’s instructions.

### ELISA

We collected supernatant samples from CHON-001 cells and measured the concentrations of secreted IL-6, IL-8, and TNF-α using ELISA kits (BD Biosciences) following the manufacturer’s instructions. Subsequently, the OD at 450 nm was measured using a Multiskan Spectrum microplate spectrophotometer (MD, USA).

### Lactate dehydrogenase (LDH) assay

We assessed the release of LDH from CHON-001 cells using an LDH Cytotoxicity Assay Kit (Sigma). Cells were cultured in 12-well plates for 48 h. Following treatment, we collected both the supernatant and total lysate from the CHON-001 cells, and these samples were incubated with the LDH reaction mixture according to the manufacturer’s instructions for 15 min. The absorbance at 490 nm was then measured, and LDH release was quantified using a microplate reader (BioTek, USA).

### Statistical analysis

Statistical analysis was performed using SPSS 20.0 software. The results are presented as the mean ± SD from three independent experiments. The statistical significance of differences among three or more groups and between two groups was assessed using one-way analysis of variance (ANOVA) or Student’s t test, respectively. *P < 0.05 and **P < 0.01 were considered to indicate statistically significant differences.

## Results

### MiR-214-3p was identified as a direct target of LINC00958

**Fig. 1:**
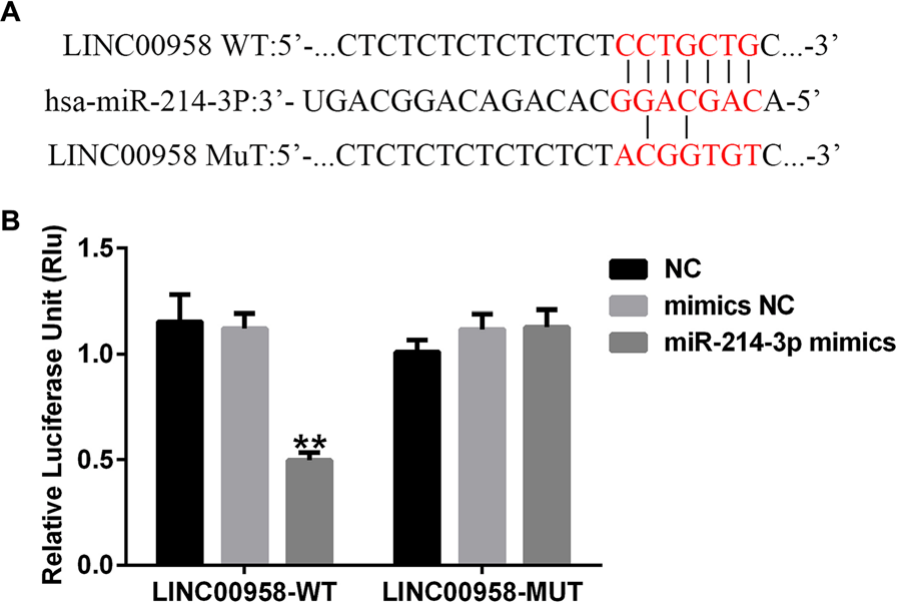
LINC00958 directly targeted miR-214-3p. **A** Schematic representation of the miR-214-3p binding site in the LINC00958 3′-UTR. **B** Relative luciferase activity was measured using a dual-luciferase reporter assay. *P < 0.05, **P < 0.01 versus NC

To investigate whether LINC00958 functions as a competing endogenous RNA by targeting miRNAs, we employed the target prediction tool StarBase to identify potential target genes. Our analysis revealed that LINC00958 and miR-214-3p may have binding sites (Fig. 1A). To further validate this interaction, we employed a dual luciferase reporter system, which confirmed that The miR-214-3p mimics decreased the activity of LINC00958 reporter gene plasmids while having no effect on mutant plasmids (Fig. 1B). This evidence strongly suggested that miR-214-3p directly interacts with LINC00958.

### Expression of LINC00958 and miR-214-3p in articular cartilage tissue samples from OA patients and in IL-1β-stimulated CHON-001 cells

**Fig. 2:**
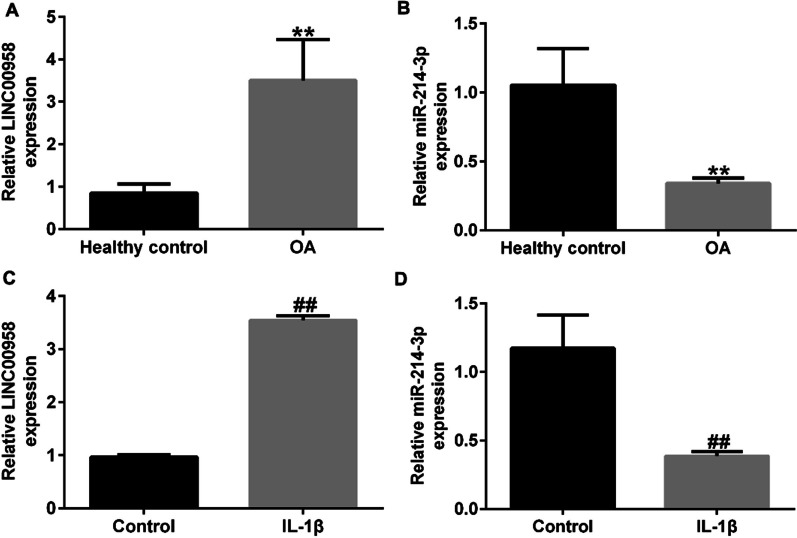
LINC00958 was upregulated and miR-214-3p was downregulated in OA patients and IL-1β-stimulated CHON-001 cells. **A**, **B** qRT-PCR analysis of LINC00958 and miR-214-3p expression in articular cartilage tissue samples from OA patients. **C**, **D** The levels of LINC00958 and miR-214-3p in IL-1β-induced CHON-001 cells were measured by qRT-PCR assay. *P < 0.05, **P < 0.01 versus Healthy control; ^##^*P *< 0.01 versus Control

Moreover, we assessed the expression levels of LINC00958 and miR-214-3p in articular cartilage tissue samples obtained from OA patients. qRT‒PCR analysis revealed upregulation of LINC00958 in the articular cartilage tissues of OA patients, as shown in Fig. 2A, in contrast to the normal control group. Furthermore, as demonstrated in Fig. 2B, the expression level of miR-214-3p was significantly lower in the articular cartilage tissues from OA patients than in those from normal control individuals. Additionally, we examined the expression of LINC00958 and miR-214-3p in an in vitro model of chondrocyte inflammatory injury induced by IL-1β. Our findings revealed upregulation of LINC00958 and downregulation of miR-214-3p in IL-1β-stimulated CHON-001 cells (Fig. 2C, D). These observations confirmed the involvement of both LINC00958 and miR-214-3p in the progression of OA.

### LINC00958 negatively regulated the expression of miR-214-3p in CHON-001 cells

**Fig. 3:**
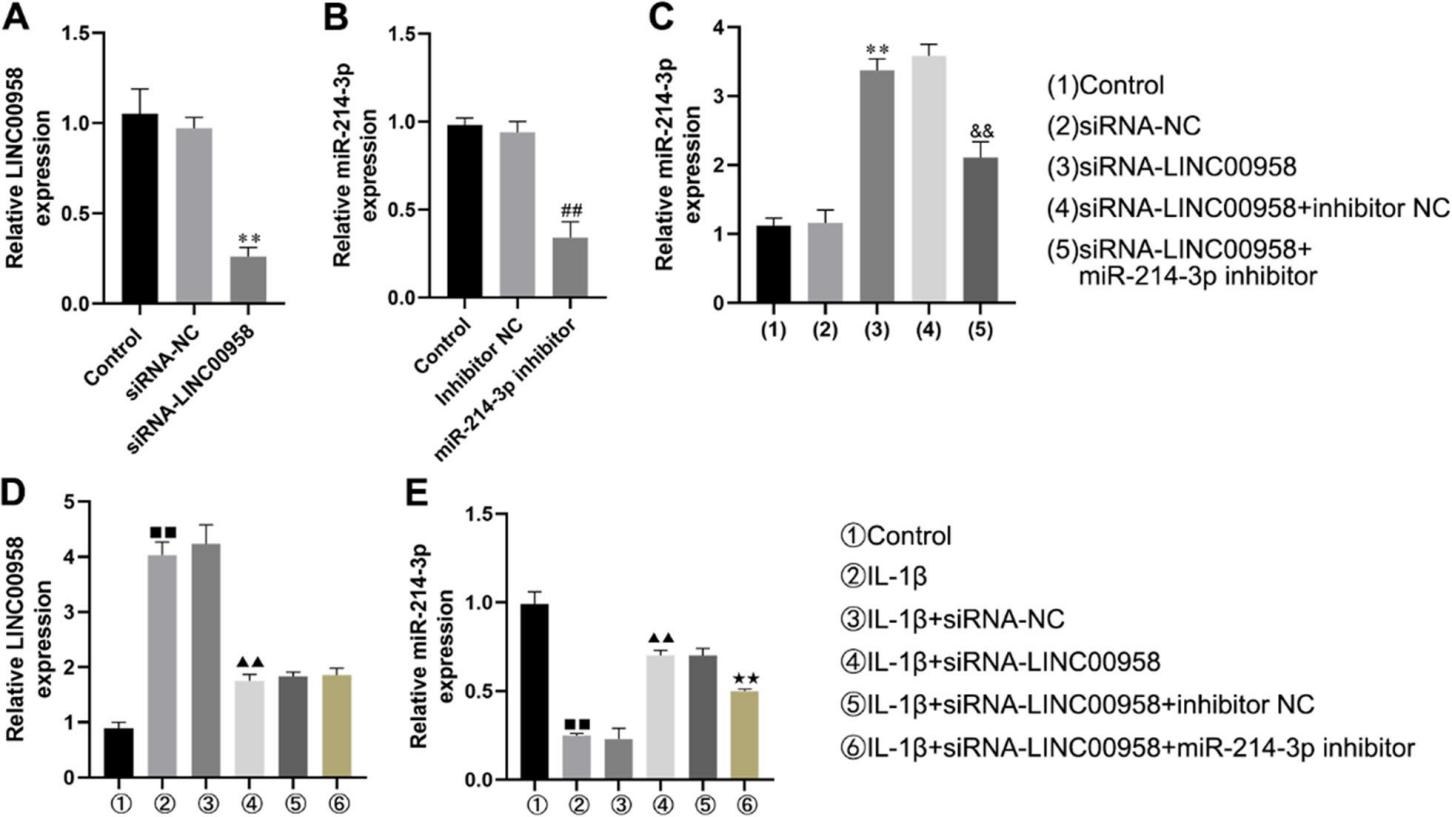
Knockdown of LINC00958 reduced LINC00958 expression and increased miR-214-3p expression. CHON-001 cells were stimulated with 10 ng/ml IL-1β, and transfected with control-siRNA, the miR-214-3p inhibitor, the inhibitor control or LINC00958-siRNA. **A**–**E** The levels of LINC00958 and miR-214-3p were measured using qRT-PCR. **P < 0.01 versus siRNA-NC group; ^##^*P* < 0.01 versus Inhibitor NC group; ^&&^*P* < 0.01 versus siRNA-LINC00958 + inhibitor NC group. ^■■^*P* < 0.01 versus Control group; ^▲▲^*P* < 0.01 versus IL-1β + siRNA-NC group; ^★^P < 0.05, ^★★^*P* < 0.01 versus IL-1β + siRNA-LINC00958 + inhibitor NC group

We then assessed the functional roles of LINC00958 and miR-214-3p in CHON-001 cells. These cells were stimulated with 10 ng/ml IL-1β and transfected with various constructs, including control-siRNA, the miR-214-3p inhibitor, the inhibitor control, and LINC00958-siRNA. The transfection efficiency was determined by qRT‒PCR. As shown in Fig. 3A, the introduction of LINC00958-siRNA led to a substantial reduction in LINC00958 expression in CHON-001 cells. In contrast, the miR-214-3p level was significantly lower in cells transfected with the miR-214-3p inhibitor than in cells in the control, control-siRNA, and inhibitor control groups (Fig. 3B).

Additionally, LINC00958-siRNA transfection markedly increased the level of miR-214-3p in CHON-001 cells. However, this increase was effectively countered by transfection of the miR-214-3p inhibitor (Fig. 3C). These findings aligned with our earlier results, which indicated that IL-1β induced an increase in LINC00958 expression and a decrease in miR-214-3p expression and that these effects were reversed by LINC00958-siRNA transfection. Furthermore, we detected the opposite results in cells transfected with the miR-214-3p inhibitor, as evidenced by the upregulation of LINC00958 expression and the downregulation of miR-214-3p expression (Fig. 3D, E). Taken together, these findings provide strong evidence that LINC00958 exerts a negative regulatory effect on miR-214-3p expression in CHON-001 cells.

### Downregulation of LINC00958 alleviated the decrease in the viability and increase in the apoptosis in IL-1β-stimulated CHON-001 cells by targeting miR-214-3p

**Fig. 4:**
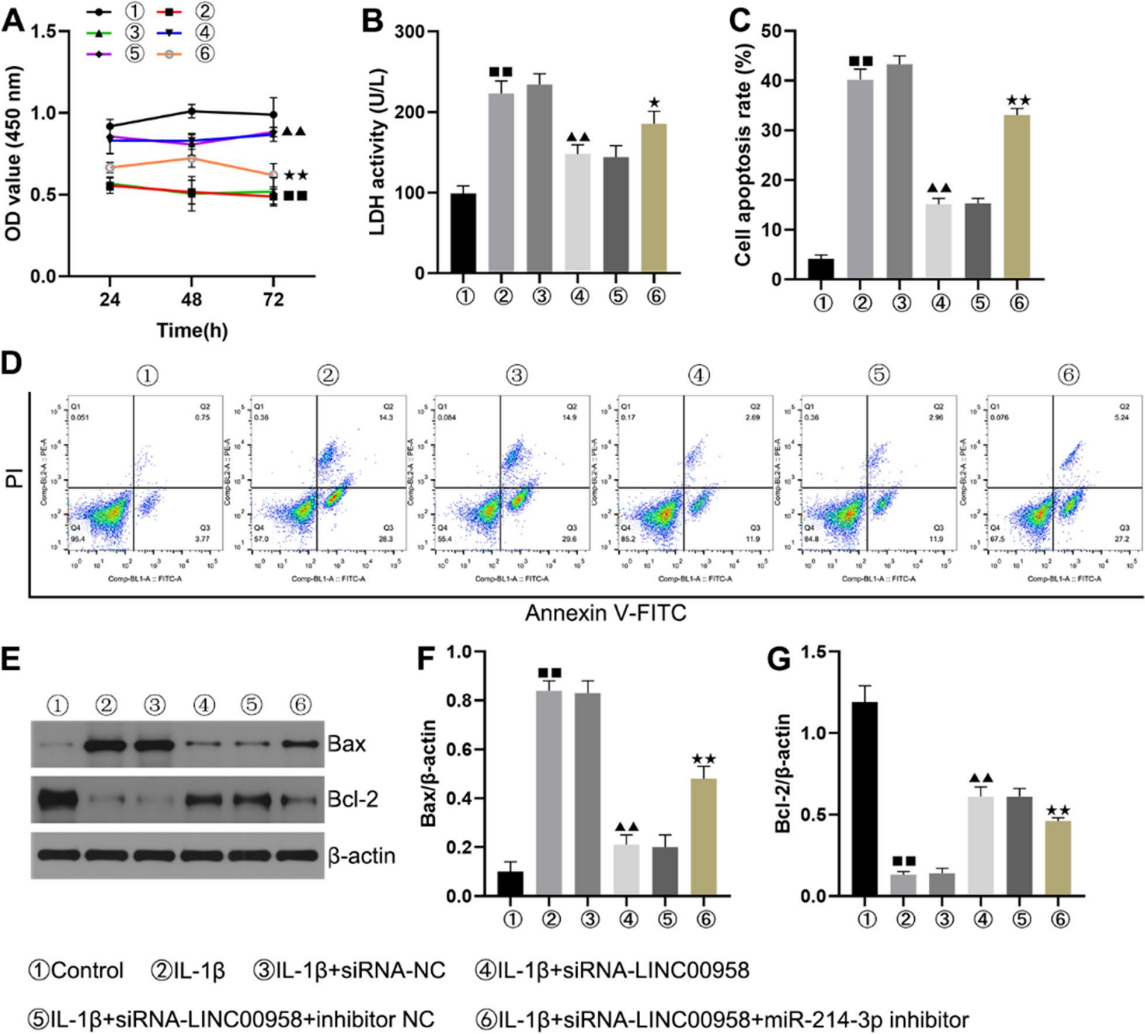
Knockdown of LINC00958 inhibited IL-1β-induced chondrocyte apoptosis by upregulating miR-214-3p. **A** Cell viability was evaluated by an MTT assay. **B** LDH release was measured to evaluate cell injury. **C** Quantification of apoptotic CHON-001 cells. **D** Flow cytometry analysis of CHON-001 cell apoptosis. **E**–**G** The protein levels of BAX and BCL2 were measured by Western blotting. ^■■^*P* < 0.01 versus Control group, ^▲▲^*P* < 0.01 versus IL-1β + siRNA-NC group, ^★^*P* < 0.05, ^★★^*P* < 0.01 versus IL-1β + siRNA-LINC00958 + inhibitor NC group

To elucidate the roles of LINC00958 and miR-214-3p in regulating the viability and apoptosis of CHON-001 cells, we stimulated these cells with 10 ng/ml IL-1β for 12 h. Additionally, we transfected cells with control-siRNA, LINC00958-siRNA, the inhibitor control, or the miR-214-3p inhibitor. Exposure to IL-1β led to a reduction in cell viability (Fig. 4A), an increase in LDH release (Fig. 4B), increases in apoptotic cell populations (Fig. 4C, D), an increase in BAX expression (Fig. 4E, F), and inhibition of BCL2 expression (Fig. 4E, G). We observed the opposite effects in cells transfected with LINC00958-siRNA. Importantly, these effects were consistently reversed by transfection of the miR-214-3p inhibitor, highlighting the potential of LINC00958 downregulation to mitigate the IL-1β-induced reduction in cell viability and increase in apoptosis in CHON-001 cells and indicating that LINC00958 achieves this modulatory effect by targeting miR-214-3p.

### Downregulation of LINC00958 alleviated the IL-1β-induced release of inflammatory factors from CHON-001 cells

**Fig. 5:**
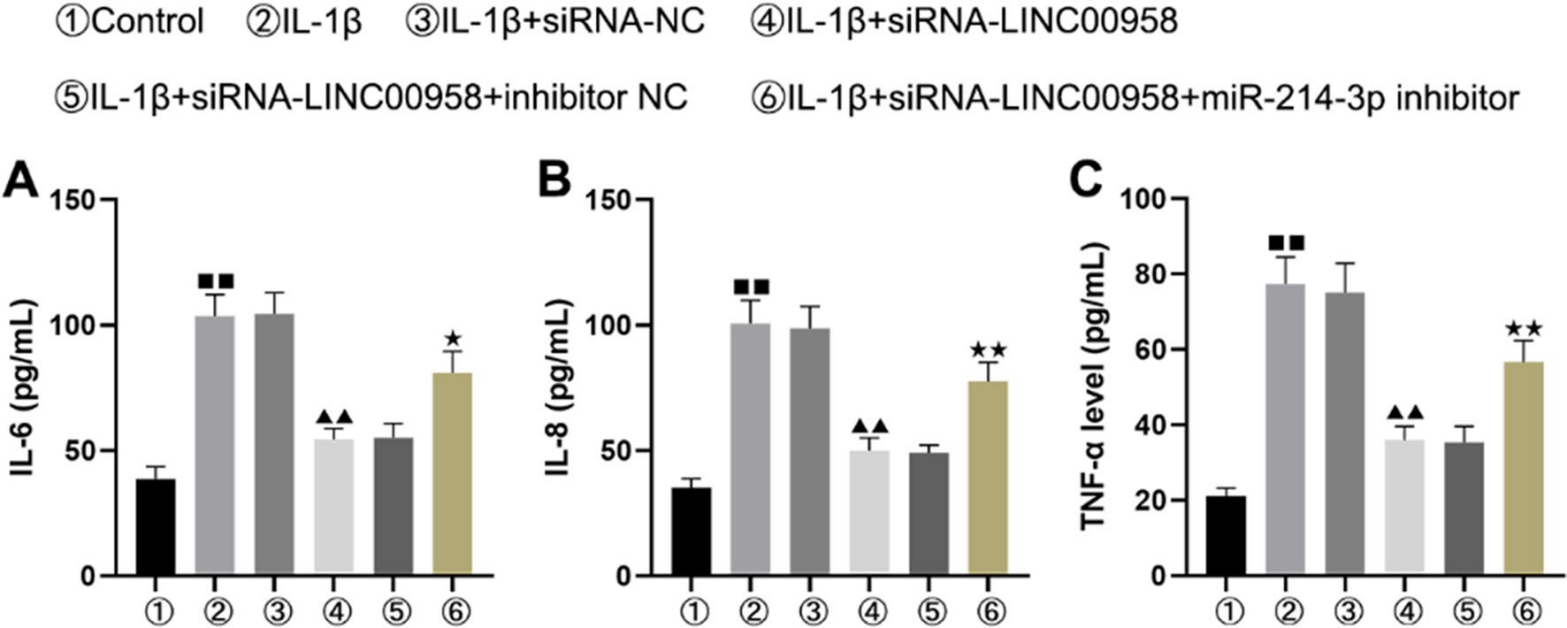
Knockdown of LINC00958 inhibited IL-1β-induced inflammatory injury in chondrocytes by upregulating miR-214-3p. The secretion of IL-6 (**A**), IL-8 (**B**) and TNF-α (**C**) was evaluated by ELISA. ^■■^*P* < 0.01 versus control group, ^▲▲^*P* < 0.01 versus IL-1β + siRNA-NC group, ^★^*P* < 0.05, ^★★^*P* < 0.01 versus IL-1β + siRNA-LINC00958 + inhibitor NC group

Furthermore, we elucidated the impacts of LINC00958 and miR-214-3p on the inflammatory response in CHON-001 cells by specifically measuring IL-6 (Fig. 5A), IL-8 (Fig. 5B), and TNF-α (Fig. 5C) concentrations. Our ELISA results indicated significant increases in the concentrations of these inflammatory factors in IL-1β-treated CHON-001 cells. Importantly, introduction of LINC00958-siRNA significantly inhibited this inflammatory response compared to that in the control-siRNA group.

However, these inhibitory effects were subsequently reversed following treatment with the miR-214-3p inhibitor. These findings indicated that downregulation of LINC00958 effectively mitigated the IL-1β-induced inflammatory response in CHON-001 cells.

### MiR-214-3p mimic transfection alleviated the decrease in the viability and increase in the apoptosis of IL-1β-induced CHON-001 cells

**Fig. 6:**
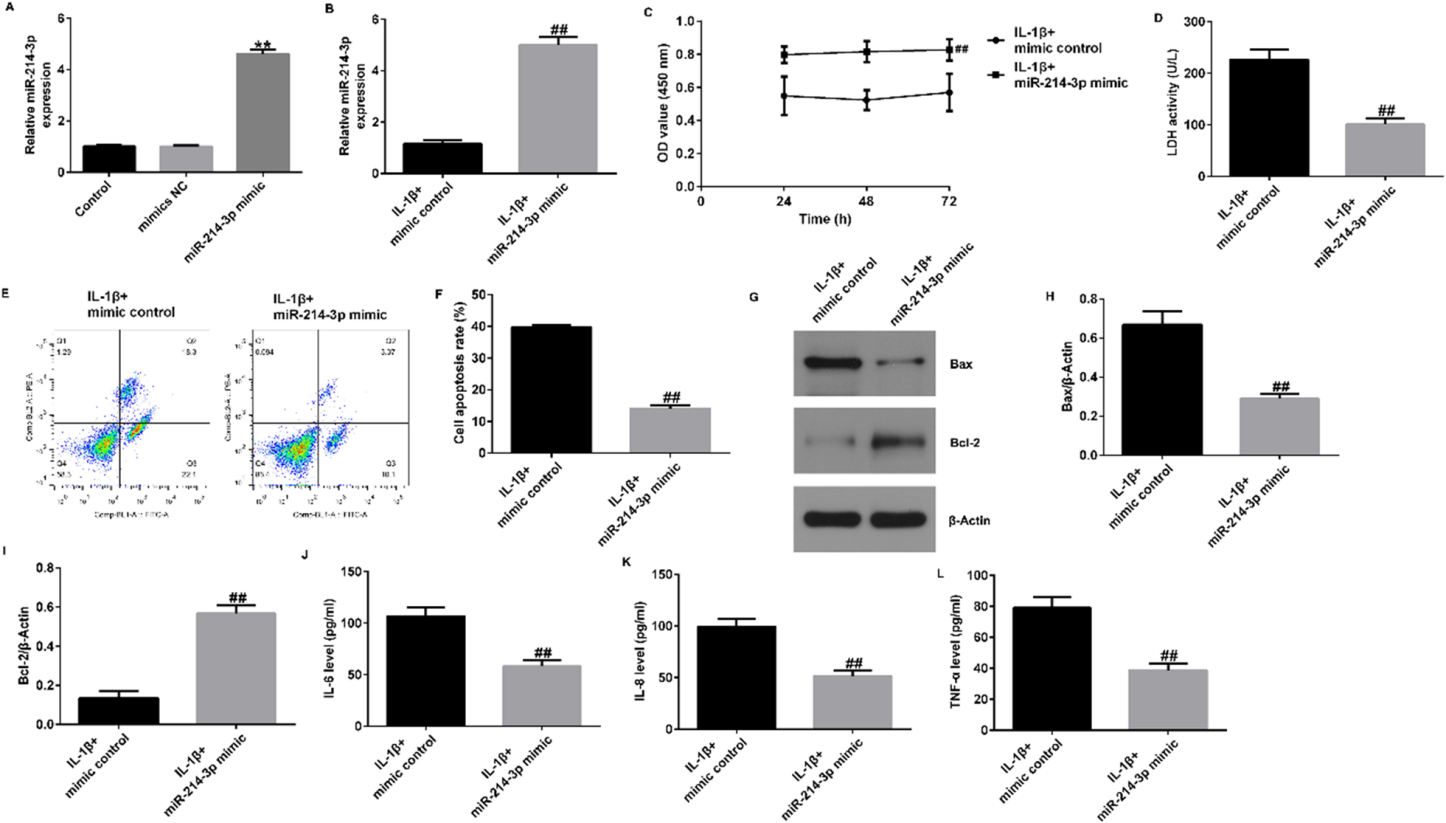
MiR-214-3p inhibited IL-1β-induced inflammatory damage in chondrocytes. CHON-001 cells were stimulated by 10 ng/ml IL-1β for 12 h and transfected with mimic control or miR-214-3p mimic. **A**, **B** measurement of the miR-214-3p level using qRT-PCR. **C** MTT assay of cell viability. **D** LDH release was measured to evaluate cell injury. **E** Cellular apoptosis was evaluated by FCM. **F** Quantification of apoptotic cells. **G** Western blotting analysis of BAX and BCL2 protein expression. qRT-PCR analysis of BAX (**H**) and BCL2 (**I**) mRNA expression in the different groups. The release of IL-6 (**J**), IL-8 (**K**) and TNF-α (**L**) was quantified using ELISA. *P < 0.05, **P < 0.01 versus control group; ^##^*P* < 0.01 versus IL-1β + mimic control group

To gain further insight into the effects of miR-214-3p in IL-1β-stimulated CHON-001 cells, we stimulated cells with 10 ng/ml IL-1β for 12 h. Subsequently, we transfected either the mimic control or the miR-214-3p mimic into the cells. As shown in Figs. 4A and 6B, miR-214-3p was markedly upregulated in the miR-214-3p mimic group compared to the control and mimic control groups.

As shown by our MTT and LDH release assays, transfection of the miR-214-3p mimic significantly augmented cell viability (Fig. 6C) while decreasing LDH release (Fig. 6D). Additionally, upregulation of miR-214-3p resulted in suppression of apoptosis (Fig. 6E and F), reduced BAX expression (Fig. 6G and H), and an increase in BCL2 expression (Fig. 6G and I). These findings indicate that the miR-214-3p mimic effectively alleviated the IL-1β-induced decrease in the viability and increase in the apoptosis of CHON-001 cells.

### Upregulation of miR-214-3p relieved IL-1β-treated inflammatory response in CHON-001 cells

Similarly, we investigated the impact of the miR-214-3p mimic on the release of inflammatory factors from CHON-001 cells. Our ELISA results indicated significant reductions in the secretion of IL-6, IL-8, and TNF-α from CHON-001 cells treated with the miR-214-3p mimic (Fig. 6J–L). Collectively, these findings strongly suggest that the miR-214-3p mimic alleviated the inflammatory response induced by IL-1β in CHON-001 cells.

### MiR-214-3p negatively regulated FOXM1 expression in CHON-001 cells by targeting FOXM1

**Fig. 7:**
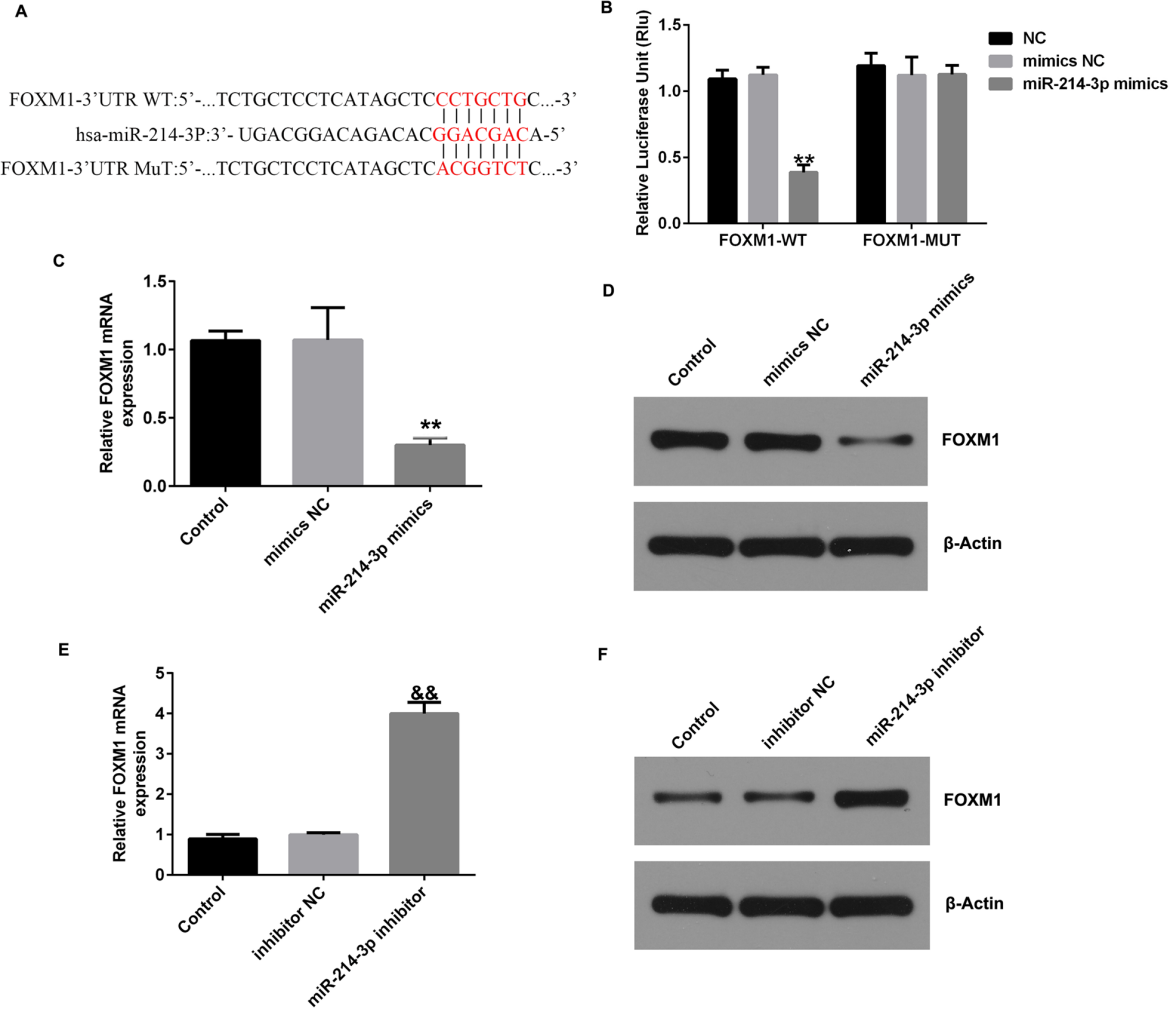
MiR-214-3p was found to be involved in OA by regulating FOXM1 expression. **A** The putative miR-214-3p binding sites in FOXM1 are shown. **B** The dual-luciferase reporter assay results confirmed the interaction between miR-214-3p and FOXM1. qRT-PCR analysis of miR-214-3p mRNA expression in miR-214-3p mimic-transfected (**C**) and miR-214-3p inhibitor-transfected (**E**) cells. Measurement of the miR-214-3p level in miR-214-3p mimic-transfected (**D**) or miR-214-3p inhibitor-transfected (**F**) cells by western blotting. **P < 0.01, versus mimics NC group; ^&&^*P* < 0.01 versus inhibitor NC group

Next, we elucidated the potential mechanisms involving miR-214-3p in CHON-001 cells. Utilizing the online database starBase, we identified a binding site for miR-214-3p in FOXM1 (Fig. 7A). Subsequently, a dual-luciferase reporter system was used to validate the interaction between miR-214-3p and FOXM1. Notably, transfection of the FOXM1 mimic significantly reduced the luciferase activity in the miR-214-3p-WT group, while no evident changes were observed in the miR-214-3p-MUT group (Fig. 7B). Furthermore, western blot and qRT‒PCR analyses revealed that the FOXM1 level was elevated in cells transfected with the miR-214-3p mimic but markedly reduced upon miR-214-3p inhibition (Fig. 7C–F). Collectively, these results substantiate the hypothesis that silencing LINC00958 impedes OA progression through the miR-214-3p/FOXM1 axis.

## Discussion

OA is a prevalent joint ailment characterized by articular cartilage degeneration and deterioration, and is a significant global public health concern. Accumulating evidence implicates various factors, including factors such as mechanical stress, structural abnormalities, and obesity, in the aetiology of OA [2, 17]. As the global population ages, the incidence of OA continues to greatly increase annually. Recently, traditional Chinese medicinal compounds have garnered attention for their potential use in OA treatment, owing to their anti-inflammatory properties and limited side effects [18]. However, a definitive OA treatment remains elusive, promoting our research to explore novel and effective therapeutic strategies for OA management.

LncRNAs have emerged as pivotal players in the pathogenesis of numerous diseases, including OA [19–21]. Ji et al. [22] revealed the regulatory role of the lncRNA BLACAT1 in modulating the differentiation of bone marrow stromal stem cells by targeting miR-142-5p in OA. Moreover, LINC00958 has been implicated in various cancer types, including bladder cancer [23], breast cancer [24] and hepatocellular carcinoma [25]. Despite these findings, the specific function of LINC00958 in the context of OA remains unclear. Thus, our research focused on revealing the mechanistic role of LINC00958 in OA. Furthermore, accumulating investigations have revealed the involvement of lncRNAs in disease pathogenesis through their interactions with miRNAs. Initially, we identified the target miRNA of LINC00958 and confirmed the direct interaction between LINC00958 and miR-214-3p. To shed light on the roles of LINC00958 in OA, we examined the expression levels of LINC00958 and miR-214-3p in articular cartilage tissue samples from OA patients. Our findings revealed upregulation of LINC00958 and downregulation of miR-214-3p in articular cartilage tissues of OA patients compared to those of normal control volunteers. Compelling evidence highlights the pivotal role of excessive IL-1β production in arthritic joints, which is closely linked to the onset and progression of OA, through the regulation of chondrocyte apoptosis and inflammatory responses [4]. In our study, we established an in vitro OA model by stimulating CHON-001 cells with 10 ng/ml IL-1β for 12 h. Our data consistently indicated that LINC00958 was upregulated, while miR-214-3p was downregulated in IL-1β-induced CHON-001 cells, coonsistent with previous reports [26]. These findings collectively suggest that LINC00958 may contribute to OA progression by modulating miR-214-3p expression.

Numerous reports have highlighted the important roles played by lncRNAs in various biological functions, including cell viability, apoptosis, and metastasis [27, 28]. Subsequently, we performed on functional analyses with LINC00958-siRNA or miR-214-3p inhibitor to elucidate the mechanism through which they mediate IL-1β’s effects on CHON-001 cells. In our experiments, CHON-001 cells were stimulated with 10 ng/ml IL-1β and subsequently transfected with control-siRNA, the miR-214-3p inhibitor, the inhibitor control, or LINC00958-siRNA. Our results were reproducibly consistent with previous findings, confirming that LINC00958 exerts a negative regulatory effect on miR-214-3p expression in CHON-001 cells. Furthermore, the impact of IL-1β was observed as it led to diminished cell viability and an increase in LDH release. Bcl-2, which is localized primarily in the cytoplasm, exerts its anti-apoptotic effect via targeting to the nucleus. Conversely, Bax, another crucial mediator, diminishes the anti-apoptotic effect of Bcl-2, ultimately leading to apoptotic cell death [29]. Our examination of Bax and Bcl-2 expression in CHON-001 cells revealed that IL-1β stimulation amplified BAX expression while concurrently inhibiting BCL-2 expression. Intriguingly, when LINC00958-siRNA was introduced, we observed contrasting results, specifically a decrease in BAX expression and an increase in BCL-2 expression. Notably, these changes were entirely reversed following miR-214-3p inhibitor transfection. Collectively, these findings illustrate that silencing LINC00958 may mitigate the IL-1β-induced reduction in CHON-001 cell viability and the induction of apoptosis by modulating miR-214-3p expression.

Inflammatory processes also play a pivotal role in driving the progression of OA, contributing to the degradation of joint tissues. Notably, Fu conducted research indicating that hesperidin protects against inflammation induced by IL-1β in human OA chondrocytes [30]. To clarify this observation, we investigated the secretion of inflammatory cytokines from IL-1β-induced CHON-001 cells, focusing on IL-6, IL-8, and TNF-α. Our ELISA results revealed that downregulation of LINC00958 effectively mitigated the inflammatory response provoked by IL-1β in CHON-001 cells. Consequently, suppressing chondrocyte apoptosis and alleviating the inflammatory response might offer valuable therapeutic benefits in OA.

To further investigate specific roles of miR-214-3p in OA, we initially stimulated CHON-001 cells with 10 ng/ml IL-1β for 12 h, and then transfected them with either the mimic control or miR-214-3p mimic. Our subsequent functional assays yielded insightful results revealed that upregulation of miR-214-3p had alleviated the IL-1β-induced decrease in the viability and increase in the apoptosis of CHON-001 cells. This effect was evidenced by the increased cell viability and reduced LDH release. Furthermore, we observed that transfection of the miR-214-3p mimic led to suppression of apoptosis, a reduction in BAX expression, and an increase in BCL-2 expression. To further investigate these findings, we also assessed the impact of the miR-214-3p mimic on the release of inflammatory cytokines. ELISA demonstrated significant reduction in the secretion of IL-6, IL-8, and TNF-α from miR-214-3p mimic-treated CHON-001 cells. Taken together, these results strongly suggest that the miR-214-3p mimic effectively alleviated the IL-1β-induced inflammatory response in CHON-001 cells. Finally, we investigated the potential mechanisms involving miR-214-3p in CHON-001 cells. Utilizing the online database StarBase and a dual-luciferase reporter system, we successfully confirmed the interaction between miR-214-3p and FOXM1. Further Western blotting and qRT-PCR analyses revealed that miR-214-3p mimic transfection increased the FOXM1 level, whereas inhibition of miR-214-3p had the opposite effect, shedding light on the regulatory role of miR-214-3p in this context.

Building upon the aforementioned research, we found that silencing LINC00958 effectively inhibited the progression of OA. This inhibition was achieved primarily through the mitigation of apoptosis and the suppression of the inflammatory response in IL-1β-stimulated CHON-001 cells, which were mediated primarily through the miR-214-3p/FOXM1 axis. Consequently, LINC00958 emerged as a promising candidate therapeutic biomarker in OA. To gain a more comprehensive understanding of the precise role of LINC00958 in the development of OA, additional in vivo experiments that can elucidate the exact underlying mechanisms are needed.

## Data Availability

The dataset used and/or analyzed in this study is available from the corresponding author upon reasonable request.
